# Regulation of TRPV5 transcription and expression by E2/ERα signalling contributes to inhibition of osteoclastogenesis

**DOI:** 10.1111/jcmm.13718

**Published:** 2018-07-31

**Authors:** Tengfei Song, Tao Lin, Jun Ma, Lei Guo, Ling Zhang, Xuhui Zhou, Tianwen Ye

**Affiliations:** ^1^ Department of Orthopaedic surgery Shanghai Changzheng Hospital Second Military Medical University Shanghai China; ^2^ Shanghai Key Laboratory for Bone and Joint Diseases Shanghai Institute of Orthopaedics and Traumatology Shanghai Ruijin Hospital Shanghai Jiaotong University School of Medicine Shanghai China; ^3^ Department of Medical Genetics Second Military Medical University shanghai China

**Keywords:** NF‐κB, oestrogen, oestrogen Receptor α, ostoeclasts, TRPV5

## Abstract

The increasing of osteoclasts formation and activity because of oestrogen (E2) deficiency is very important in the aetiology of postmenopausal osteoporosis. Our previous studies showed that E2 inhibited osteoclastic bone resorption by increasing the expression of Transient Receptor Potential Vanilloid 5 (TRPV5) channel. However, the exact mechanism by which E2 increases TRPV5 expression is not fully elucidated. In this study, Western blot, quantitative real‐time PCR, tartrate‐resistant acid phosphatase staining, F‐actin ring staining, chromatin immunoprecipitation and luciferase assay were applied to explore the mechanisms that E2‐induced TRPV5 expression contributes to the inhibition of osteoclastogenesis. The results showed that silencing or overexpressing of TRPV5 significantly affected osteoclasts differentiation and activity. Silencing of TRPV5 obviously alleviated E2‐inhibited osteoclastogenesis, resulting in increasing of bone resorption. E2 stimulated mature osteoclasts apoptosis by increasing TRPV5 expression. Further studies showed that E2 increased TRPV5 expression through the interaction of the oestrogen receptor α (ERα) with NF‐κB, which could directly bind to the fragment of −286 nt ~ −277 nt in the promoter region of *trpv5*. Taken together, we conclude that TRPV5 plays a dominant effect in E2‐mediated osteoclasts formation, bone resorption activity and osteoclasts apoptosis. Furthermore, NF‐κB plays an important role in the transcriptional activation of E2‐ERα stimulated TRPV5 expression.

## 1 INTRODUCTION

Oestrogen (E2) plays an important role in the control of bone mass and bone strength. The decrease in E2 produces osteoporosis in most postmenopause women. E2 loss at menopause leads to enhanced bone turnover with the increase in bone‐forming by osteoblasts and even greater rate of bone resorption by osteoclasts, which result in loss of predominantly trabecular bone.^1^, ^2^ Both of osteoblasts and osteoclasts are confirmed to respond to E2.^3^ However, the increasing of osteoclasts formation and bone resorption activity by E2 deficiency is recognized as a central role in postmenopausal osteoporosis (PMOP).^4^, ^5^

Osteoclasts arise from hematopoietic stem cells that, in the presence of receptor activator of nuclear factor κB (RANK) ligand (RANKL) and macrophage‐colony stimulating factor (MCS‐F), undergo differentiation and fusion resulting in large multinucleated cells.^6^, ^7^ Postmenopause E2 withdrawal increase the production of proinflammatory cytokines, such as IL‐1, IL‐6 and tumour necrosis factor (TNF)‐α from stroma, monocytes and lymphoid cells, which could promote the differentiation of myeloid precursor cells into osteoclasts resulting bone resorption.^8^ The alternate, E2 could also act directly on osteoclast precursors to inhibit its differentiation, but its mechanism is not fully elucidated.^9^ It is well‐known that E2 binding to oestrogen receptor (ER) directly regulates cells physiological effects through genomic and non‐genomic mechanisms. In genomic response, E2/ER binding to specific genes in the nucleus affects their transcription, resulting in de novo protein synthesis. In non‐genomic response, E2 elicits rapid responses independently of genome interaction and protein synthesis.^10^ The precise molecular events underlying the effect of E2 on osteoclasts differentiation need to be further explored.

Calcium ion (Ca2+) signals are involved in osteoclast differentiation and bone resorption. RANKL‐evoked [Ca2+]i oscillations play an important role in osteoclast differentiation, which could stimulate osteoclast‐specific gene expression through activation of the nuclear factor of activated T cells, cytoplasmic, calcineurin‐dependent 1 (NFATc1) pathway.^11^, ^12^ Osteoclast differentiation is dependent not only on intracellular Ca2+ release but also on extracellular Ca2+ influx. Transient Receptor Potential Vanilloid 5 (TRPV5) of TRP superfamily has clarified the nature of the calcium entry channels. Our previous studies showed that silencing of TRPV5 could alleviate E2‐decreased osteoclasts differentiation and bone resorption.^13^, ^14^ However, the exact mechanisms by which E2‐mediated TRPV5 expression contributes to the inhibition of osteoclastogenesis are not fully elucidated.

In this study, we found that the depletion of TRPV5 significantly increased osteoclastogenesis and alleviated E2‐inhibited osteoclasts formation. In contrast, overexpression of TRPV5 could inhibit RANKL‐induced osteoclasts formation and enhance the inhibitory effect of E2 on osteoclast differentiation. Silencing of TRPV5 significantly alleviated E2‐induced osteoclasts apoptosis. Furthermore, silencing of ERα, but not ERβ, could significantly reduce the stimulatory effect of E2 on TRPV5 expression, suggesting that E2 up‐regulated TRPV5 expression through ERα. A further study shows that the region between −500 nt and −150 nt on the *trpv5* promoter contains regulatory elements, and it is critical for the transcription regulation of *trpv5* by E2‐ERα. In addition, we found that E2 increased TRPV5 expression through the interaction of the ERα with NF‐κB, which could directly bind to the fragment of −286 nt ~ −277 nt in the promoter region of *trpv5*. Based on these results, we believe that TRPV5 plays a dominant effect in E2‐mediated osteoclast formation, bone resorption activity and osteoclasts apoptosis. Furthermore, NF‐κB plays an important role in the transcriptional activation of E2‐ERα stimulated TRPV5 expression.

## 2 MATERIALS AND METHODS

### 2.1 Cell culture

Murine Raw264.7 cells line was supplied by Shanghai Institute of Orthopaedics and Traumatology. Raw264.7 cells were cultured with alpha‐minimal essential media (α‐MEM) (Invitrogen, Paisley, UK) supplemented with 10% foetal bovine serum and 100 μg/mL penicillin/streptomycin. Raw264.7 cells were cultured with complete α‐MEM medium containing RANKL (50 ng/mL) (Peprotech, Rocky Hill, NJ) for 7 days to acquire mature osteoclasts.

Primary bone marrow‐derived macrophages (BMMs) were isolated from the long bones of 5‐week‐old C57BL/6J mice as previous reports.^15^ Briefly, cells isolated from the bone marrow of femur and tibiae were cultured in a 100‐mm dish with complete α‐MEM medium supplemented with 10 ng/mL M‐CSF for 24 hours. Non‐adherent cells were harvested to culture with fresh medium containing 50 ng/mL M‐CSF for 3 days. Then the adherent cells were harvested as osteoclasts precursors. These cells were then seeded and further cultured with complete α‐MEM medium containing M‐CSF (30 ng/mL) and RANKL (50 ng/mL) for 7 days to acquire mature osteoclasts.

### 2.2 Lentiviral transduction and oligonucleotide transfection

Raw264.7 cells or osteoclasts precursors were inoculated into 6‐well tissue culture plates at a density of 1 × 10^5^ cells in α‐MEM medium. Once cells reached approximately 70% confluence, cells were infected with TRPV5 shRNA lentiviral particles, TRPV5 lentiviral activation particles, ERα shRNA lentiviral particles or ERβ shRNA lentiviral particles (Santa Cruz, CA, USA) for 24 hours. Furthermore, control cells were transduced with control lentiviral activation particles (Mock) and/or control shRNA lentiviral particles‐A (Santa Cruz, CA, USA). Subsequently, the medium was replaced with fresh α‐MEM containing 50 ng/mL RANKL and 10^−7^ M E2 (Sigma‐Aldrich, St Louis, MO, USA) for primary culture. Small interfering siRNA duplexes targeting AP‐1, SP1 and NF‐κB and corresponding negative control (NC) were purchased from Cell signaling Technology (Beverly, CA, USA). Raw264.7 cells were treated with 10^−7^ M E2 and transfected with siRNA using Lipofectamine 3000 (Invitrogen, Paisley, UK).

### 2.3 Rhodamine phalloidin and tartrate‐resistant acid phosphatase (TRAP) staining

Rhodamine phalloidin staining was accomplished as previous studies,^15^ which was applied to demonstrate Raw264.7 cells differentiation to the active form of osteoclasts. Briefly, the cells were fixed in 4% paraformaldehyde, followed by incubation with 0.1% Triton X for 5 minutes. Then, the solution was replaced with rhodamine phalloidin solution, and cells were kept stationary in a dark room for 30 minutes. Confocal laser scanning microscopy (Carl Zeiss, Oberkochen, Germany) was used to detect the fluorescence signal. The number of osteoclasts with a fluorescent ring was counted. For TRAP staining, TRAP solution was added to the well and incubated with the cells at 37°C for 15 min. The number of cells with 3 or more nuclei was counted under an optical microscope.

### 2.4 TRAP activity assessment

TRAP activity was quantified by a colorimetric method as previous studies.^15^ Briefly, cells grown in 48‐well plates were washed with PBS and incubated at 37°C with 200 μL mixture containing 0.1% SDS, 2 mg p‐nitrophenol phosphate, acetate and tartrate solution (Sigma) for 30 minutes. The reaction was quenched by adding 40 μL of 0.5 mol/L NaOH, and absorbance was read at 405 nm.

### 2.5 Pit formation assay

Bone resorption activity was assessed by pit formation assay as previous reports.^16^ Briefly, Raw264.7 cells were cultured on bovine cortical bone slices in 24‐well plates and induced by RANKL for 7 days. The slices were then placed for 10 minutes in 1 mol/L NH4OH and were sonicated to remove the cells. The cell‐free slices were stained in 1% toluidine blue in 1% sodium borate for three minutes. Three view fields were randomly selected for each bone slice for further analysis. The percentage of resorbed bone surface area was counted using the Image J software. Experiments were repeated independently at least three times.

### 2.6 Measurement of caspase‐3 activity

Caspase‐3 activity was measured as previous studies.^17^ The protein samples were prepared as indicated in western blot analysis. Then, 50 mg of total proteins was added to the reaction buffer containing Ac‐DEVD‐pNA (2 mmol/L), incubated for 2 hours at 37°C, and the absorbance of yellow pNA cleaved from its corresponding precursors was measured using a spectrometer at 405 nm. The specific caspase‐3 activity, normalized for total proteins of cell, was then expressed as fold of the baseline caspase activity of control cell.

### 2.7 quantitative real‐time PCR (qRT‐PCR)

Total RNA was isolated from cells using Trizol reagent (Invitrogen, Carlsbad, CA) according to the manufacturer's instruction. Next, cDNA was synthesized from 1 μg of total RNA using reverse transcriptase (TaKaRa Biotechnology, Japan). QRT‐PCR was performed to amplify the cDNA using the SYBR Premix Ex Tag kit (TaKaRa Biotechnology, Japan) and an ABI 7500 Sequencing Detection System (Applied Biosystems, Foster City, CA, USA). The mouse primer sequences for *TRPV5* (Accession Numbers: NM_001007572), *ER*α (Accession Numbers: NM_007956), *ER*β (Accession Numbers: NM_010157), *TRAP* (Accession Numbers: NM_011611), *c‐fos* (Accession Numbers: NM_010234), *Cathepsin K* (Accession Numbers: NM_ 007802), *DC‐STAMP* (Accession Numbers: NM_001289513), *V‐ATPase* α*3* (Accession Numbers: NM_016921), *V‐ATPase d2* (Accession Numbers: NM_175406) and β*‐actin* (Accession Numbers: NM_007393) are described in Table 1.

**Table 1:** Premiers for qRT‐PCR analysis

Gene	Forward Premier (5′‐3′)	Reverse Premier (5′‐3′)
*TRPV5*	ATGGGGGCTAAAACTCCTTGG	CCTCTTTGCCGGAAGTCACA
*ER*α	CCTCCCGCCTTCTACAGGT	CACACGGCACAGTAGCGAG
*ER*β	CTGTGATGAACTACAGTGTTCCC	CACATTTGGGCTTGCAGTCTG
*TRAP*	CACTCCCACCCTGAGATTTGT	CATCGTCTGCACGGTTCTG
*c‐fos*	CGGGTTTCAACGCCGACTA	TTGGCACTAGAGACGGACAGA
*Cathepsin K*	GAAGAAGACTCACCAGAAGCAG	TCCAGGTTATGGGCAGAGATT
*DC‐STAMP*	GGGGACTTATGTGTTTCCACG	ACAAAGCAACAGACTCCCAAAT
*V‐ATPase a3*	CACAGGGTCTGCTTACAACTG	CGTCTACCACGAAGCGTCTC
*V‐ATPase d2*	CAGAGCTGTACTTCAATGTGGAC	AGGTCTCACACTGCACTAGGT
β*‐actin*	GGCTGTATTCCCCTCCATCG	CCAGTTGGTAACAATGCCATGT

### 2.8 ChIP

A ChIP assay was performed using the EZ‐ChIPTM kit (Millipore, Billerica, MA) according to the manufacturer's instructions. The following antibodies were utilized to immunoprecipitate cross linked protein–DNA complexes: rabbit anti‐ NF‐κB p65 (D14E12, Cell signaling Technology, Beverly, CA, USA) and normal rabbit IgG (12‐370, Millipore, Billerica, MA).

### 2.9 Western blot analysis

Proteins from cell lysates were prepared in 1× sodium dodecyl sulphate buffer, separated by sodium dodecylsulphate‐polyacrylamide gel electrophoresis (SDS‐PAGE) and transferred to a nitrocellulose membrane (Bio‐Rad, Hercules, CA, USA). The membranes were blocked with 5% fat‐free milk and incubated with the appropriate antibody. Antigen–antibody complex was detected with enhanced chemiluminescence reagents (Pierce, Rockford, IL,USA). Quantification of the immunoblots was performed using Image J software. The optical density values obtained for TRPV5, ERα, ERβ were normalized to density values acquired for GAPDH. The optical density values obtained for Bcl2 and Bax were normalized to density values acquired for GAPDH. The Western blot analysis and the calculation of the relative quantity of protein were conducted on three independent preparations.

### 2.10 Luciferase reporter assay

Raw264.7 cells were seeded in 96‐well plates at a density of 5000 cells per well. After 24 hours, the cells were transiently transfected with a mixture of 5 ng of pRL‐CMV Renilla luciferase reporter, 50 ng of the firefly luciferase reporter and 5 pmol small RNA (siRNAs). After treating E2 48 hours, luciferase activity was measured using the dual‐luciferase reporter assay system (Promega, Madison, WI, USA).

### 2.11 Statistical analysis

Data were collected from three or more independent experiments and expressed as mean ± SD. A two‐sided Student's *t*‐test was used to analyse the difference between groups. One‐way analysis of variance (ANOVA) was performed to show the difference between groups.

## 3 RESULTS

### 3.1 Role of TRPV5 in osteoclasts differentiation

**Figure 1:**
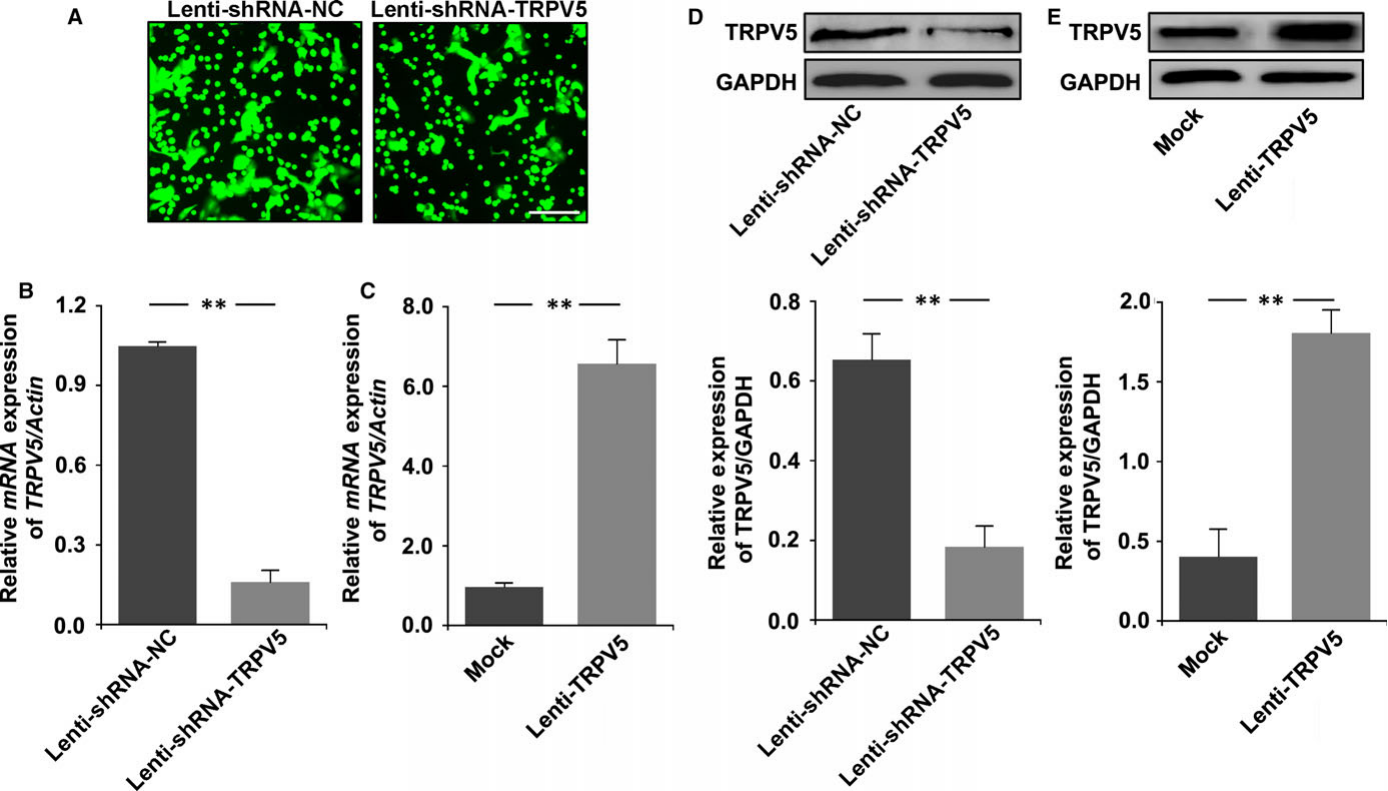
Effective depletion/overexpression of TRPV5 by Lenti‐shRNA‐TRPV5/Lenti‐TRPV5 in Raw264.7 cells. A, All cells expressed GFP, showing that cells were infected by lentivirus. Scale bars = 20 μm. B, Verified TRPV5 knockdown effect by lentivirus‐mediated transduction of Raw264.7 cells. ShRNAs was showed to deplete 80% of the expression of *TRPV5* in Raw264.7 cells by real‐time RT‐PCR. n* = *4, **P < .01. C, Verified overexpressed effect by lentivirus‐mediated transduction of Raw264.7 cells. Real‐time RT‐PCR analysis showed that *TRPV5* expression was up‐regulated about seven folds in Raw264.7 cells infected with Lenti‐TRPV5. n* = *4, **P < .01. D, Western blot analysis showed that the depletion of TRPV5 was effective in Raw264.7 cells infected with Lenti‐shRNA‐TRPV5. n* = *4, **P < .01. E, Western blot analysis showed that TRPV5 expression was up‐regulated about four folds in Raw264.7 cells infected with Lenti‐TRPV5. n* = *4, **P < .01

**Figure 2:**
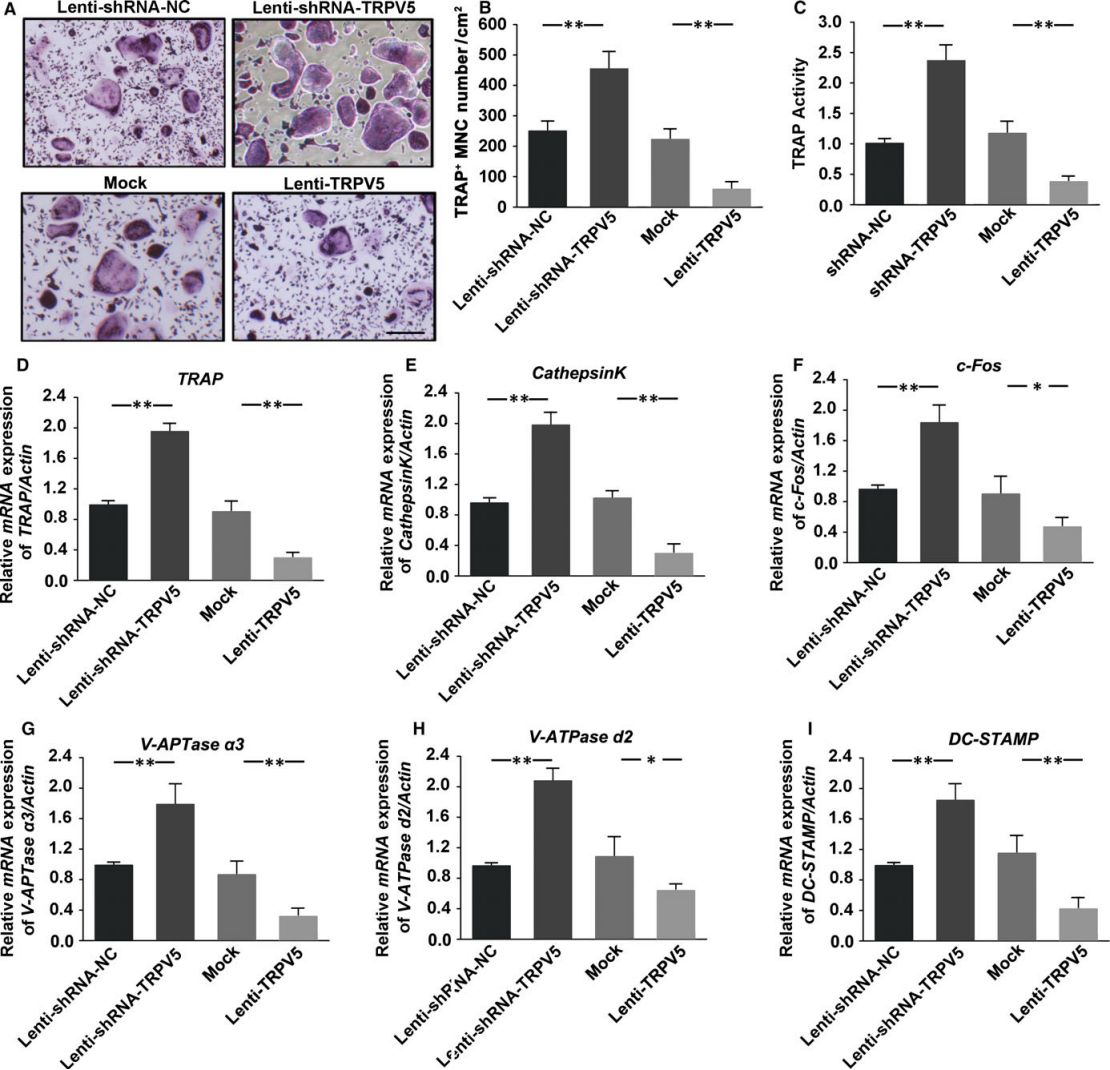
TRPV5 is involved in osteoclasts differentiation. (A) TRAP staining was showed in Raw 264.7 cells infected with either Lenti‐shRNA‐TRPV5 or Lenti‐TRPV5 under 50 ng/mL RANKL for 7 days. Scale bars = 20 μm. (B) Summarized data showed that depletion of TRPV5 significantly increased osteoclastogenesis. However, overexpression of TRPV5 significantly decreased osteoclasts differentiation. n* = *6, **P < .01. (C) TRAP activity assessment was accomplished in Raw 264.7 cells infected with either Lenti‐shRNA‐TRPV5 or Lenti‐TRPV5 under 50 ng/mL RANKL for 7 days. The results showed that the TRAP activity was significantly increased in TRPV5‐depleted osteoclasts. In contrast, overexpression of TRPV5 inhibited TRAP activity. n* = *4, **P < .01. (D‐I) QRT‐PCR analysis of osteoclasts formation specific genes, *TRAP* (D), *CathepsinK* (E), *c‐Fos* (F), *V‐ATPase* α*3* (G)*, V‐ATPase d2* (H) and *DC‐STAMP* (I) in Raw 264.7 cells infected with either Lenti‐shRNA‐TRPV5 or Lenti‐TRPV5 under 50 ng/mL RANKL for 7 days. The results showed that these genes expressions were obviously up‐regulated in TRPV5‐depleted osteoclasts, whereas they were markedly suppressed in TRPV5‐overexpressed osteoclasts. n* = *4, **P < .01, *P < .05

To delineate the role of TRPV5 in the differentiation from pre‐osteoclasts to mature osteoclasts, the level of TRPV5 was decreased with lentiviral constructs encoding shRNA targeting TRPV5 (Lenti‐shRNA‐TRPV5) or increased with lentiviral constructs encoding TRPV5 (Lenti‐TRPV5). For lentivirus‐mediated TRPV5 expression, optimal viral particle numbers for infection were based on infection efficiency, determined from the percentage of target cells with green fluorescent protein (GFP). All cells expressed GFP, showing that cells were infected by lentivirus (Figure 1A). QRT‐PCR and Western blot analysis of TRPV5 expression confirmed that the silencing and overexpression of TRPV5 were effective (Figure 1B‐E). Next, we observed the effect of TRPV5 on osteoclasts formation. Raw264.7 cells were infected with Lenti‐shRNA‐TRPV5 or Lenti‐TRPV5 under 50 ng/mL RANKL for 7 days. The results of TRAP staining showed that the number of mature osteoclasts significantly increased in TRPV5‐depleted osteoclasts. In contrast, overexpression of TRPV5 inhibited osteoclasts formation (Figure 2A, B). Being consistent with the results of TRAP staining, TRPV5 also affected TRAP activity of osteoclasts (Figure 2C). Many genes have been identified to be associated with osteoclasts differentiation, such as *TRAP*,* Cathepsink*,* c‐Fos*,* DC‐STAMP*,* V‐ATPase* α*3* and *V‐ATPase d2*. To further examine the effect of TRPV5 on osteoclasts formation, we observed the effects of TRPV5 on these genes expressions. The results showed that these genes expressions were obviously up‐regulated in TRPV5‐depleted osteoclasts, whereas they were markedly suppressed in TRPV5‐overexpressed osteoclasts (Figure 2D‐I). These results suggested that TRPV5 was involved in the process of osteoclasts formation.

### 3.2 The effects of TRPV5 on osteoclastic bone resorption and F‐actin ring formation

**Figure 3:**
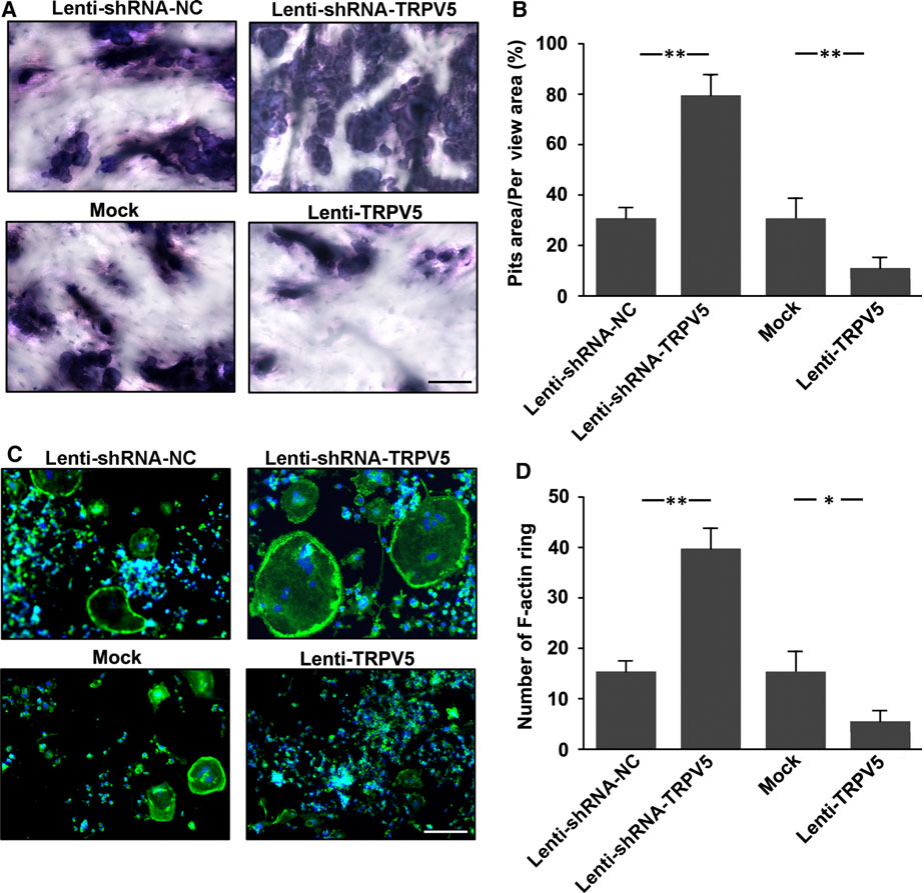
The effects of TRPV5 on osteoclastic bone resorption and F‐actin ring formation. A, Resorption pit formation was showed in Raw 264.7 cells infected with either Lenti‐shRNA‐TRPV5 or Lenti‐TRPV5 under 50 ng/mL RANKL for 7 days. Scale bars = 25 μm. B, Summarized data showed that depletion of TRPV5 significantly increased resorption pit formation by osteoclasts. However, overexpression of TRPV5 significantly decreased resorption pit formation by osteoclasts. n* = *6, **P < .01. C, F‐actin ring staining was performed to estimate the effect of TRPV5 on osteoclastic bone resorption. Scale bars = 20 μm. D, Summarized data showed depletion of TRPV5 significantly increased the number of F‐actin ring. However, overexpression of TRPV5 significantly decreased the number of F‐actin ring. n* = *4, **P < .01, *P < .05

To further investigate the effect of TRPV5 on osteoclast function, pit formation assay was firstly applied to observe the effect of TRPV5 on osteoclastic bone resorption. Raw264.7 cells were cultured on bone slices, and infected with Lenti‐shRNA‐TRPV5 and Lenti‐TRPV5 in the presence of 50 ng/mL RANKL for 7 days. We found a significant increase in pit formation in TRPV5‐silenced osteoclasts. However, the resorption area markedly decreased in TRPV5‐overexpressed group (Figure 3A, B). Furthermore, a well‐polarized F‐actin ring is required for efficient bone resorption. Therefore, we performed F‐actin ring staining to estimate the effect of TRPV5 on osteoclastic bone resorption. We found that clear F‐actin ring structures and increased number of F‐actin ring were observed in the Lenti‐shRNA‐TRPV5 group compared with Lenti‐shRNA‐NC group (Figure. 3C). The F‐actin ring structure was significantly disrupted in TRPV5‐overexpressed osteoclasts (Figure. 3D). We found that the increase in the pit area and actin rings mediated by treatment of Lenti‐shRNA‐TRPV5 was apparently because of an increase in multinuclear osteoclasts formed in the cultures. Similarly, the inhibition of the pit and actin rings formation by Lenti‐TRPV5 was apparently because of a reduction in multinuclear osteoclasts formed in the cultures. Thus, we considered that TRPV5 affected osteoclastic bone resorption by attenuating osteoclastogenesis, resulting in less mature osteoclasts, and as a consequence, to that reduced bone resorption.

### 3.3 Silencing of TRPV5 alleviates E2‐inhibited osteoclast formation and bone resorption

**Figure 4:**
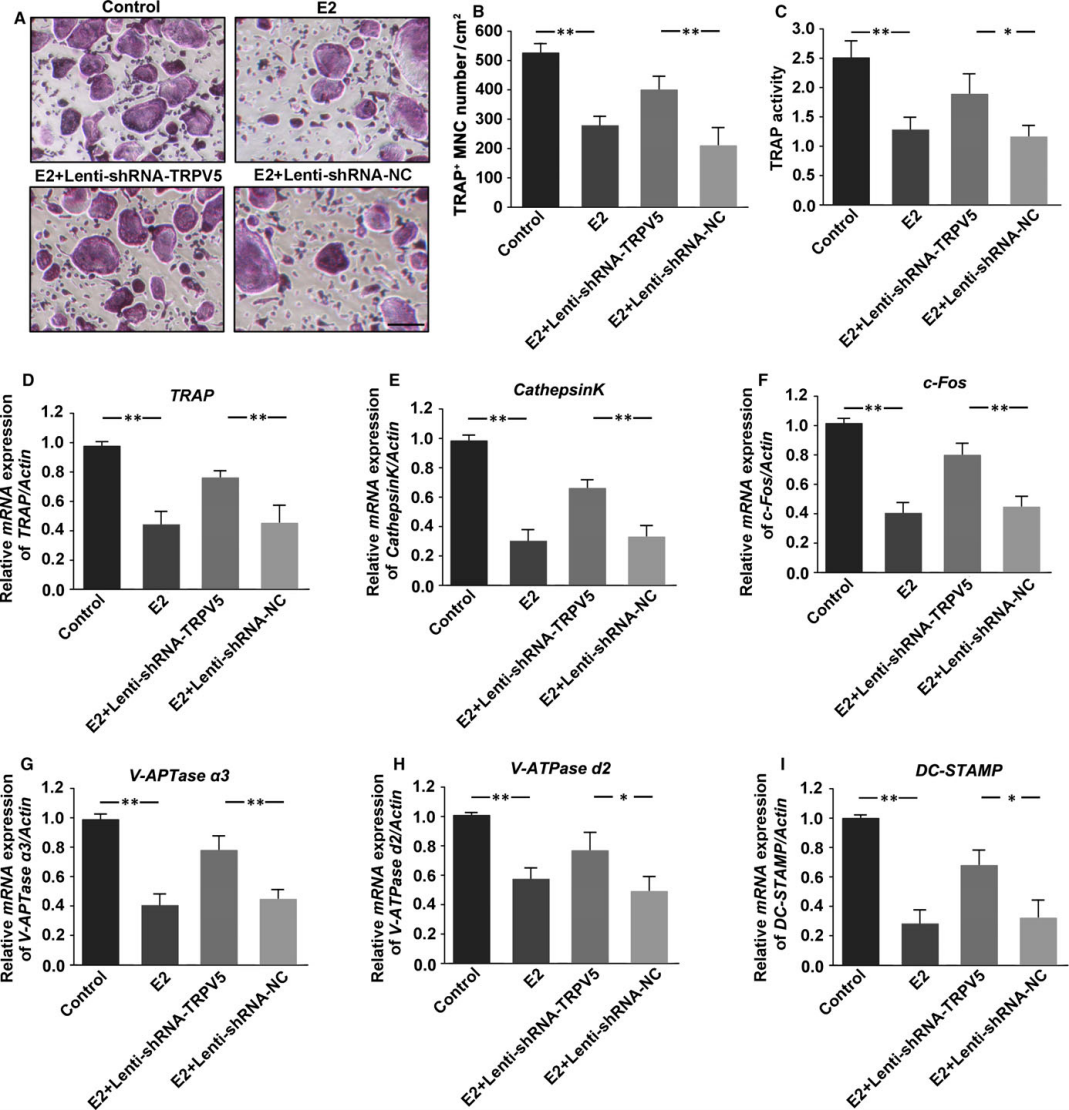
Silencing of TRPV5 alleviates E2‐inhibited osteoclast formation. (A) TRAP staining was showed in Raw 264.7 cells treated with 10^−7^ M E2 and infected with either Lenti‐shRNA‐TRPV5 or Lenti‐TRPV5 under 50 ng/mL RANKL for 7 days. Scale bars = 20 μm. (B) Summarized data showed that depletion of TRPV5 significantly alleviated E2‐decreased osteoclastogenesis. n* = *6, **P < .01. (C) TRAP activity assessment was accomplished in Raw 264.7 cells treated with 10^−7^ M E2 and infected with either Lenti‐shRNA‐TRPV5 or Lenti‐TRPV5 under 50 ng/mL RANKL for 7 days. n* = *4, **P < .01, *P < .05. (D‐I) QRT‐PCR analysis of osteoclasts formation specific genes, *TRAP* (D), *CathepsinK* (E), *c‐Fos* (F), *V‐ATPase* α*3* (G)*, V‐ATPase d2* (H) and *DC‐STAMP* (I) in Raw 264.7 cells treated with 10^−7^ M E2 and infected with either Lenti‐shRNA‐TRPV5 or Lenti‐TRPV5 under 50 ng/mL RANKL for 7 days. The results showed that the expressions of these specific genes for osteoclasts formation were decreased in osteoclasts treated with E2, whereas they were also attenuated by silencing of TRPV5. n* = *4, **P < .01, *P < .05

**Figure 5:**
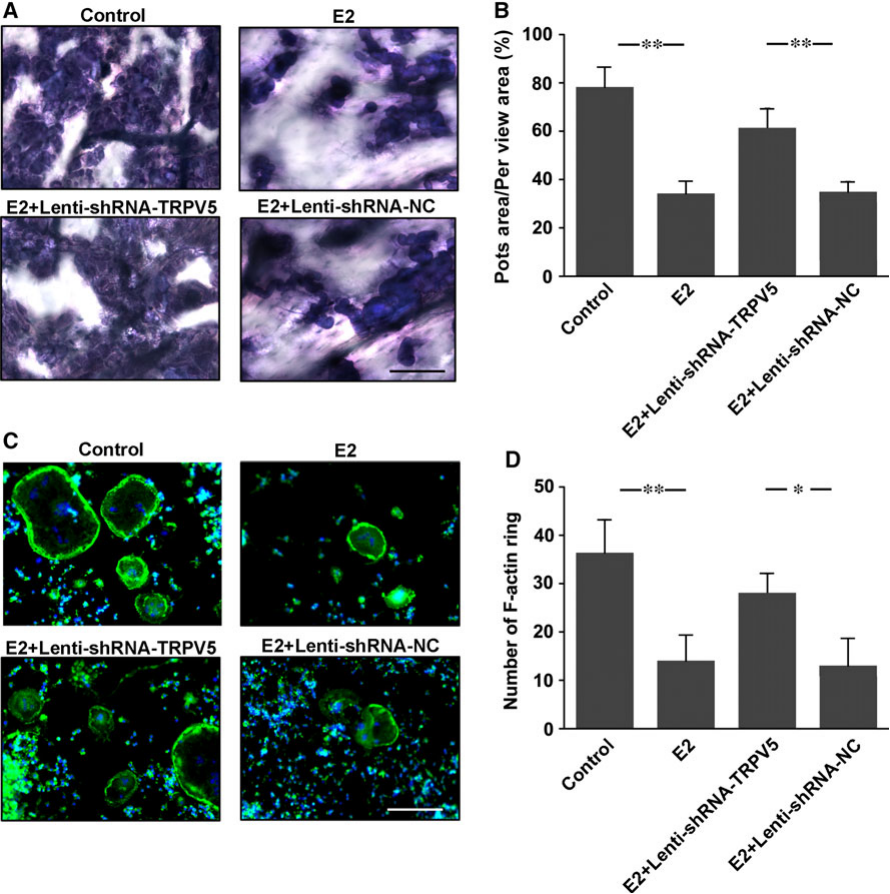
Silencing of TRPV5 alleviates E2‐inhibited osteoclastic bone resorption and F‐actin ring formation. A,Resorption pit formation was showed in Raw 264.7 cells treated with 10^−7^ M E2 and infected with Lenti‐shRNA‐TRPV5 under 50 ng/mL RANKL for 7 days. Scale bars = 25 μm. B, Summarized data showed that depletion of TRPV5 significantly alleviated E2‐decreased resorption pit formation by osteoclasts. n* = *6, **P < .01. C, F‐actin ring staining was performed to estimate the effect of TRPV5 on E2‐inhibited osteoclastic bone resorption. Scale bars = 20 μm. D, Summarized data showed depletion of TRPV5 significantly alleviated E2‐decreased the number of F‐actin ring. n* = *4, **P < .01, *P < .05

To further examine the effect of TRPV5 on E2‐inhibited osteoclasts formation, TRAP staining was applied in osteoclasts infected with Lenti‐shRNA‐TRPV5 under 10^−7^ M E2 and 50 ng/mL RANKL for 7 days. The results showed that E2 significantly inhibited osteoclasts formation, which was alleviated by silencing of TRPV5 (Figure 4A, B). Being consistent with the results of TRAP staining, TRPV5 also attenuated E2‐inhibited TRAP activity of osteoclasts (Figure 4C). Similarly, the expressions of specific genes for osteoclasts formation were decreased in osteoclasts treated with E2, whereas they were also attenuated by silencing of TRPV5, suggesting that TRPV5 was involved in E2‐inhibited osteoclast formation (Figure 4D‐I). Next, we applied pit formation assay to observe the effect of TRPV5 on E2‐inhibited osteoclastic bone resorption. The results showed that pit formation was obviously decreased in the osteoclasts treated with E2. However, the inhibitory effect of E2 on osteoclastic bone resorption was markedly alleviated in TRPV5‐silenced osteoclasts (Figure 5A, B). Similarly, the number of clear F‐actin ring structures was decreased in the E2‐treated group compared with control group, whereas it was alleviated in TRPV5‐silenced osteoclasts treated with E2 (Figure 5C, D). These results implicated that TRPV5 was involved in E2‐inhibited osteoclastic bone resorption.

### 3.4 E2 promotes osteoclasts apoptosis by increasing TRPV5 expression

**Figure 6:**
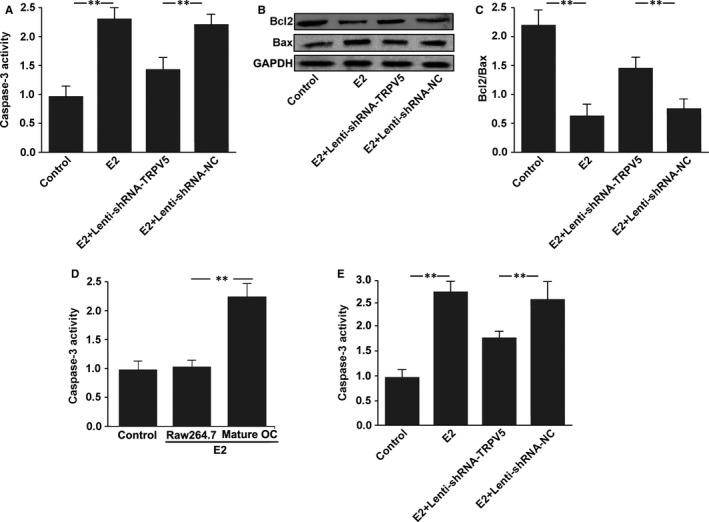
E2 promotes osteoclasts apoptosis by increasing TRPV5 expression. A, The effects of E2 and Lenti‐shRNA‐TRPV5 on caspase‐3 activity in osteoclasts. The results showed that caspase‐3 activity was significantly increased in mature osteoclasts treated with 10^−7^ M E2 for 12 h, whereas it could be reduced by silencing of TRPV5. n* = *4, **P < .01. B, The levels of Bcl‐2 and Bax protein from osteoclasts treated with E2 and Lenti‐shRNA‐TRPV5 were analysed by Western blot. C, Quantitative analysis of Bcl2/Bax. The Bcl‐2/Bax ratio was decreased in osteoclasts treated with 10^−7^ M E2 for 12 h. However, silencing of TRPV5 significantly alleviated the inhibitory effect of E2 on Bcl‐2/Bax ratio in osteoclasts. n* = *3, **P < .01. D, Caspase‐3 activities were measured in RAW264.7 cells and mature osteoclasts treated with 10^−7^ M E2 for 12 h. The results showed that E2 increased caspase‐3 activity in mature osteoclasts but not in RAW264.7 cells. n* = *4, **P < .01. E, The effects of E2 and Lenti‐shRNA‐TRPV5 on caspase‐3 activity in primary cultured osteoclasts. n* = *4, **P < .01

To investigate whether TRPV5 was also involved in E2‐induced osteoclasts apoptosis, we silenced TRPV5 expression using Lenti‐shRNA‐TRPV5 in osteoclast. Caspase‐3 activity assay was firstly used to examine the cells apoptosis. The results showed that caspase‐3 activity significantly increased in mature osteoclasts treated with 10^−7^ M E2 for 12 hours, whereas it was reduced by silencing of TRPV5 (Figure 6A). Because of the fact that the Bcl‐2/Bax ratio determines whether a cell will undergo apoptosis, the levels of Bcl‐2 and Bax protein from osteoclasts were analysed by Western blot. We found that E2 caused decrease in Bcl‐2 protein level and significant increase in Bax protein. The Bcl‐2/Bax ratio was decreased in osteoclasts treated with 10^−7^ M E2 for 12 hours. However, silencing of TRPV5 significantly alleviated the inhibitory effect of E2 on Bcl‐2/Bax ratio in osteoclasts (Figure 6B‐C). Taken together, these results suggested that E2 stimulated osteoclasts apoptosis by increasing TRPV5 expression. To further examine whether the effect of E2‐TRPV5 axis on the promotion of apoptosis is specific for mature osteoclasts, we assayed the caspase‐3 activity in RAW264.7 cells and mature osteoclasts treated with 10^−7^ M E2 for 12 hours. The results showed that E2 increased caspase‐3 activity in mature osteoclasts but not in RAW264.7 cells (Figure 6D). Next, we further tested the effect of TRPV5 on E2‐induced osteoclasts apoptosis in primary cultured osteoclasts. The results showed that E2 increased the caspase‐3 activity in primary cultured osteoclasts, whereas it was reduced by silencing of TRPV5 (Figure 6E).

### 3.5 ERα is involved in the regulation of TRPV5 expression by E2

**Figure 7:**
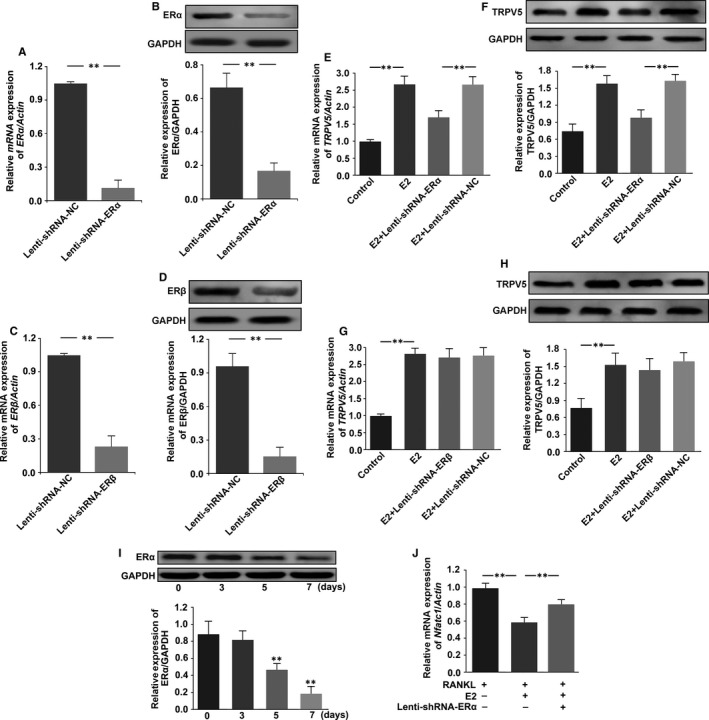
ERα is involved in the regulation of TRPV5 expression by E2. (A‐B) Verified the ERα knockdown effect by lentivirus‐mediated transduction of Raw264.7 cells. ShRNAs targeting ERα was showed to deplete about 85% of the expression of ERα in Raw264.7 cells by real‐time RT‐PCR (A) and Western blot (B). n* = 4*, **P < .01. (C‐D) Verified ERβ knockdown effect by lentivirus‐mediated transduction of Raw264.7 cells. ShRNAs targeting ERβ was showed to deplete about 80% of the expression of ERβ in Raw264.7 cells by real‐time RT‐PCR (C) and Western blot (D). n* = *4, **P < .01. (E‐F) TRPV5 expression was measured by real‐time RT‐PCR (E) and Western blot (F) in Raw 264.7 cells treated with 10^−7^ M E2 and infected with Lenti‐shRNA‐ ERα under RANKL for 48 h. n* = *4, **P < .01. (G‐H) *TRPV5* expression was measured by real‐time RT‐PCR (G) and Western blot (H) in Raw 264.7 cells treated with 10^−7^ M E2 and infected with Lenti‐shRNA‐ERβ under RANKL for 48 h. n* = *4, **P < .01. (I) ERα expression was measured by Western blot in RANKL‐induced osteoclasts differentiation. The results showed that the expression of ERα was significantly decreased during the process of osteoclasts differentiation. n* = *3, **P < .01. (J) Real‐time RT‐PCR analysis showed that blocking ERα could obviously alleviated the inhibitory effect of E2 on *NFATc1* expression. n* = *3, **P < .01

To explore the roles of two types of ER, ERα and ERβ, in E2‐increased TRPV5 expression, the level of ERα and ERβ was dividedly silenced with lentiviral constructs encoding shRNA targeting ERα (Lenti‐shRNA‐ERα) and lentiviral constructs encoding shRNA targeting ERβ (Lenti‐shRNA‐ERβ). Western blot and qRT‐PCR analysis of ERα and ERβ expression confirmed that the deletion of ERα and ERβ were effective (Figure 7A‐D). Next, the expression of TRPV5 was measured in Raw264.7 cells depleted with ERα or ERβ, followed by treating with 10^−7 ^M E2 under 50 ng/mL RANKL for 48 hours. The results of Western blot and qRT‐PCR showed that silencing of ERα significantly alleviated the stimulatory effect of E2 on TRPV5 expression. However, application of Lenti‐shRNA‐ERβ to silence ERβ did not obviously affect E2‐induced TRPV5 expression in osteoclasts (Figure 7E‐H). These results indicated that E2 regulated the expression of TRPV5 mainly through ERα. Subsequently, we measured the expression of ERα using Western blot in RANKL‐induced osteoclasts differentiation. We found that the expression of ERα was significantly decreased during the process of osteoclasts differentiation (Figure 7I). We also found that blocking ERα could obviously alleviate the inhibitory effect of E2 on NFATc1 expression (Figure 7J).

### 3.6 TRPV5 is regulated by the transcription factor NF‐κB

**Figure 8:**
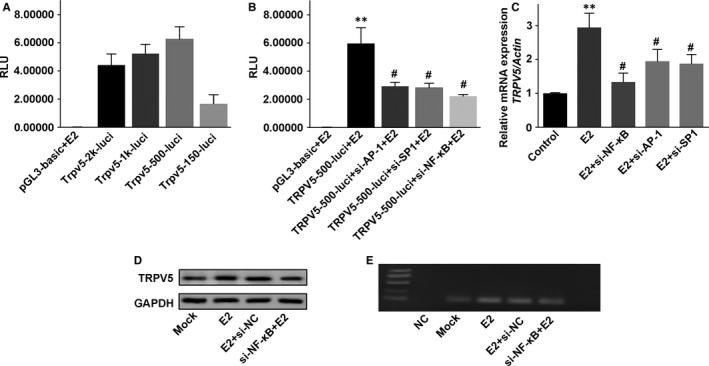
TRPV5 is regulated by the transcription factor NF‐κB. A, Serial truncations of TRPV5 promoter were cloned into pGL3‐basic vector, and these constructs were transfected into Raw264.7 cells. After that, cells were treated with E2 and luciferase activity was measured. n* = *6. B, The fragment of −500 nt ~ 0 nt in the TRPV5 promoter was cloned into luciferase reporter vector. Then Raw264.7 cells were cotransfected with luciferase reporter and siRNA against NF‐κB, AP‐1 and SP1 under 10^−7^ M E2, followed by measurement of luciferase activity. n* = *6, **P < .01 vs pGL3‐basic+E2, *^#^P < *.01 vs TRPV5‐500‐luci+E2. C, TRPV5 expressions were examined by Western blot in Raw264.7 cells treated with E2 and infected with lentiviral shRNA targeting NF‐κB, AP‐1 and SP1 under RANKL for 48 h. n* = *4, **P < .01 vs Control*, ^#^P < *.01 vs E2. D, *TRPV5 mRNA* expressions were examined by real‐time RT‐PCR in Raw264.7 cells treated with E2 and infected with lentiviral shRNA targeting NF‐κB, AP‐1 and SP1 under RANKL for 48 h. n* = *4, **P < .01. E, ChIP assay showed that NF‐κB could directly bind to the putative site on the TRPV5 promoter in Raw264.7 cells. n* = *4, **P < .01

To further explore the mechanisms by which E2 increases TRPV5 expression in osteoclasts, we applied online bioinformatical software programs JASPAR (http://jaspar.genereg.net/cgi-bin/jaspar_db.pl) to analyse promoter region of *trpv5* and the potential sites of NF‐κB, AP‐1, SP1 binding were found. Next, serial truncations of *trpv5* promoter were cloned into pGL3‐basic vector, and these constructs were transfected into Raw264.7 cells. After that, cells were treated with E2 and luciferase activity was measured. The highest activities were associated with −500 nt ~ −150 nt (Figure 8A), indicating that the fragment contained regulatory elements and it was critical for the transcription of TRPV5. Then we cotransfected luciferase reporter and siRNA against NF‐κB, AP‐1 and SP1 into Raw264.7 cells, followed by treating with E2. The results showed that NF‐κB knockdown significantly reduced luciferase activity of −500 nt ~ 0 nt fragment (Figure 8B). These results indicate that the region between −500 bp and 0 bp on the *trpv5* promoter is responsible for NF‐κB‐mediated activation of *trpv5*. To confirm TRPV5 was a transcriptional target of NF‐κB, we also measured mRNA and protein levels of TRPV5 in the case of NF‐κB, AP‐1, SP1 knockdown. The results revealed that E2 up‐regulated TRPV5 expression in Raw264.7 cells; however, it was significantly abrogated by NF‐κB knockdown (Figure 8C, D). To determine the binding site was responsive to NF‐κB‐mediated E2 resulted transcriptional activation of TRPV5, sequence analysis of −500 nt ~ −150 nt fragment uncovered the putative NF‐κB binding site located at −286 nt ~ −277 nt, chromatin immunoprecipitation (ChIP) assay was performed. Our results showed that NF‐κB directly bind to the putative site on the *trpv5* promoter in Raw264.7 cells (Figure 8E). These findings indicate that the up‐regulation of TRPV5 is mediated by NF‐κB in E2‐treated Raw264.7 cells.

## 4 DISCUSSION

Bone remodelling controls bone mass through a complex regulation of the balance between bone formation and bone resorption.^18^ It is a key factor for PMOP that E2 deficiency strongly promotes bone resorption of osteoclasts.^19^, ^20^ However, the exactly cellular and molecular mechanisms of oestrogen deficiency‐increased osteoclastic bone resorption are not fully elucidated. Here, we demonstrated that TRPV5 was a critical regulator in the osteoclastogenesis and osteoclastic bone resorption, which was also confirmed to implicate in E2‐mediated osteoclast formation, bone resorption and osteoclast apoptosis. Furthermore, E2 could up‐regulate the expression of TRPV5 mainly through ERα. Further studies showed that E2‐ERα bonding to the range of trpv5 gene promoter was the region between −500 nt and −150 nt, which included the most of the transcriptional binding sites for gene transcription and expression. In addition, NF‐κB was found to play a major role in the transcriptional activation of E2‐ERα stimulated TRPV5. This is the study linking a TRPV5 with E2‐regulated osteoclasts function and explores the mechanisms of transcriptional regulation by which E2 increase TRPV5 expression through ERα.

Previous studies showed that TRPV5 channels were highly expressed in human osteoclasts, which were located on the bone side of the plasma membrane of resorbing human osteoclasts and were consistent with the subcellular localization in rodents.^21^, ^22^ The effects of TRPV5 channels on osteoclastic bone resorption are controversial. Van der et al^22^ found that bone resorption was nearly absent in osteoclast cultures from TRPV5 knock‐out mice, supporting the TRPV5‐promoted bone resorption observed in vivo. However, Nijenhuis et al^23^ found that TRPV5 knock‐out mice developed severe hypercalciuria and reduced bone thickness, implicated that TRPV5 may has an inhibitory effect on the process of bone resorption. Chamoux et al^21^ also confirmed that TRPV5 had an inhibitory effect on the process of bone resorption in human osteoclasts. Being consistent with these studies, our results confirmed that TRPV5 inhibited osteoclastic bone resorption. In this study, the pit area and actin rings mediated by treatment of Lenti‐shRNA‐TRPV5 or Lenti‐TRPV5 were apparently because of modification of multinuclear osteoclasts formed in the cultures. Therefore, we considered that the inhibitory effect of TRPV5 on osteoclastic bone resorption was mediated through attenuation of osteoclastogenesis. Previous studies showed that E2 affected osteoclast function by attenuating osteoclastogenesis to reduce bone resorption.^9^, ^24^, ^25^ Furthermore, we found that TRPV5 was involved in E2‐decreased osteoclastogenesis and the osteoclastic bone resorption. Thus, it is likely that the inhibitory effect of TRPV5 on osteoclastic bone resorption is mediated through attenuation of osteoclastogenesis.

In addition, we further demonstrated that TRPV5 regulated osteoclasts differentiation.

E2 regulates diverse physiological effects via two pathways, the genomic pathway and the non‐genomic pathway. The genomic pathway involves DNA binding of the E2‐ER to the promoter regions of responsive genes, regulating their transcription, resulting in de novo protein synthesis.^26^ In addition, E2 can also signal through membrane receptors to elicit rapid responses, such as the activation of cAMP‐dependent protein kinase (PKA), protein kinase C, and K^+^ channel activity, resulting in the alternation of cells function.^27^ Manolagas et al^28^ suggested that nongenotropic pathway was sufficient for the bone anabolic actions of E2 with no role for classical E2 actions. However, many other studies confirmed that there was a balance between the two oestrogen signalling pathways and that the alteration of this balance had important skeletal consequences.^29^, ^30^, ^31^ Being consistent with *Syed* et al^31^ studies, our studies found that silencing of ERα alleviated E2‐increased TRPV5 expression, suggesting that genomic pathway is critical for the regulation of TRPV5 expression by E2.

The actions of E2 are mediated by two related receptors, ERα and ERβ.^32^ Vidal et al^33^ demonstrated for the first time that ERα, and not ERβ mediated the important effects of E2 on the skeleton of male mice. Sims et al^34^ found that depletion of ERβ in male mice did not have any effect on bone, whereas loss of ERα led to decrease cortical density and thickness. Furthermore, Sims et al^35^ also demonstrated that estradiol was totally ineffective in preventing orchidectomy‐induced bone loss in ERα male mice. These studies indicated that ERα plays a critical role in the regulation of bone metabolism. Being consistent with these results, we found that E2 increased the expression of TRPV5 mainly through ERα, but not ERβ. Our results further strengthen the conclusion that ERα plays a crucial role in the E2‐mediated bone metabolism.

Previous studies showed that E2 bonding to its receptors modulated target gene transcription through the classical genomic pathway and the nonclassical genomic pathway. The classical genomic pathway involves direct DNA binding of E2‐ER to oestrogen response elements (EREs)^36^ in the promoter regions of target genes.^37^ The alternate, nonclassical genomic pathway involves the indirect modulation of transcription by the interaction of the ER with other transcription complexes, such as AP‐1, SP1 and NF‐κB.^37^ Weber et al^38^ studies showed that EREs were not exist in the promoter regions of TRPV5, indicating that the classical genomic pathway may be not involved in the regulation of TRPV5 expression by E2‐liganded ER. In contrast, many studies demonstrated that the human and mouse TRPV5 promoter contained AP‐1 and Sp1 sites, which can mediate the transcriptional activation of E2‐liganded ER.^39^ Being consistent with these results, our studies confirmed that DNA binding of the E2‐ER to NF‐κB led to the increasing of TRPV5 expression, suggesting that E2 up‐regulates TRPV5 expression through the nonclassical genomic pathway. In addition, further studies demonstrated that the NF‐κB binding sites mainly located in −500 nt ~ −150 nt fragment of *trpv5* promoter.

RANKL‐evoked [Ca2 + ]i oscillations play a switch‐on role in osteoclast differentiation through the nuclear factor of activated T cells, cytoplasmic, NFATc1 activation pathway that triggers osteoclast‐specific gene expression.^11^ It was also confirmed that both Ca2 +  oscillation/calcineurin‐dependent and ‐independent signalling pathways contributed to NFATc1 activation, resulting in efficient osteoclastogenesis.^40^ Our laboratory previously demonstrated that TRPV6, which share 75% homology with TRPV5, decreased osteoclastogenesis through Ca2+ oscillation/calcineurin ‐independent signalling pathways.^41^ In the present study, we mainly explored how E2 regulated TRPV5 expression. The exact mechanisms by which up‐regulation of TRPV5 expression by E2 affected osteoclastogenesis needs to be further investigated in future studies.

In conclusion, our study provides new evidence that TRPV5 plays a dominant effect in E2‐mediated osteoclast formation, bone resorption activity and osteoclast apoptosis. Furthermore, this study further explored the molecular mechanisms by which E2 up‐regulated TRPV5 expression. The essential roles of E2‐induced TRPV5 expression in osteoclast differentiation, bone resorption and osteoclast apoptosis were described in this study, which may offer a very specific and powerful therapeutic target for treatment of bone loss caused by E2 deficiency.

## CONFLICT OF INTEREST

The authors have declared that no conflict of interest exists.

## AUTHOR CONTRIBUTIONS

All authors contributed to the design of the study. Tengfei Song, Jun Ma, Tao Lin and Lei Guo collected the data and performed the analysis. Tengfei Song led the draft of the manuscript. All authors interpreted the findings, revised the manuscript and approved the final version. Tianwen Ye and Xuhui Zhou take responsibility for the integrity of this work.
